# Fever of Unknown Origin, Wasting Syndrome and Bone Marrow Involvement: A Leprosy Case Report

**DOI:** 10.3389/fimmu.2022.916319

**Published:** 2022-07-07

**Authors:** Marcela Araujo de Oliveira Santana, Willian Vargas Tenório da Costa, Isadora Costa Celestino, Diogo Fernandes dos Santos, Bruno de Carvalho Dornelas, Matthew Martin Pavelka, Andrea De Martino Luppi, Isabela Maria Bernardes Goulart

**Affiliations:** ^1^ National Reference Center for Sanitary Dermatology and Leprosy, Clinics’ Hospital, Faculty of Medicine, Federal University of Uberlândia, Uberlândia, Brazil; ^2^ Faculty of Medicine, Federal University of Uberlândia, Uberlândia, Brazil; ^3^ Post-Graduation Program in Health Science, Faculty of Medicine, Federal University of Uberlândia, Uberlândia, Brazil; ^4^ Pathology Department, Clinical Hospital, Faculty of Medicine, Federal University of Uberlândia, Uberlândia, Brazil; ^5^ School of Medicine, Indiana University, Terre Haute, IN, United States

**Keywords:** lepromatous leprosy, bone marrow, neuropathy, fever of unknown origin, case report

## Abstract

*Mycobacterium leprae*, the etiologic agent of leprosy, is an acid-fast-staining and slow-growing bacilli that infect macrophages and Schwann cells individually or through forming globi. The clinical presentation of leprosy is broad and depends on the host immune response. We report a case of a 42-year-old Brazilian man presenting with fever of unknown origin (FUO), anemia, wasting syndrome, and neuropathy. The diagnosis of lepromatous leprosy was made after an extensive investigation revealed the presence of *M. leprae* in the bone marrow. Bone marrow involvement in leprosy is rare and some authors believe the presence of *M. leprae* in the bone marrow can act as a reservoir of the disease facilitating future relapses. It is important to investigate bone marrow involvement in leprosy, especially when the patient presents with cytopenias and positive epidemiologic history.

## Introduction

*Mycobacterium leprae*, the etiologic agent of leprosy (Hansen’s disease), is an acid-fast-staining and slow-growing bacilli (1, 2). This intracellular bacteria infects macrophages and Schwann cells individually and could grow to form globi (1, 2).

The clinical presentation of leprosy is broad and depends on the host immune response (3). It usually involves the peripheral nervous system and skin, in addition to mucous membranes in some instances (3). The presentation of leprosy related to bone marrow infiltration is rare with only a few case reports in the literature diagnosing leprosy by bone marrow biopsy or aspiration (4–6).

We report a case of a patient presenting with fever of unknown origin (FUO), anemia, wasting syndrome, and neuropathy. The investigation revealed the presence of *M. leprae* in the bone marrow, and the diagnosis of lepromatous leprosy was made.

## Case Description

A 42-year-old Brazilian man was admitted to the hospital due to wasting syndrome, fever of unknown origin (FUO), hyporexia, asthenia, and night sweats. The patient reported worsening of his condition during the last month to the point of becoming bedridden. He also reported edema up to his knees, skin peeling, and severe, bilateral lower extremity weakness.

One year before admission, the patient started presenting pain in his lower limbs that worsened during physical activities. Five months later, he started complaining of paresthesia and burning sensations from his knees to his feet. He reported no chronic conditions or significant past medical history and denied the use of any medications, drugs, alcohol, or tobacco. The family reported that his mother and brother were diagnosed with leprosy one year before the patient’s presentation.

The physical exam revealed weight loss of approximately 25 kg (55.11 lbs.) in five months, hepatosplenomegaly and lymphadenopathy confirmed with ultrasound, hypoesthesia to touch, and painful stimuli. Additional physical findings included the presence of bilaterally thickened neural trunks, inability to fully flex the fingers, and dactylitis. During the neurological exam, normal reflexes, motor coordination, and cranial nerve reactions were observed. The visual inspection yielded diffuse skin infiltration all over the body (especially on the face, hands, feet, and ears) and the presence of small lepromas on the posterior thighs and legs.

The laboratory exams revealed microcytic and hypochromic anemia (**Table 1**) and a normal pattern for serum protein electrophoresis (SPEP). Testing for HIV, T-Spot.TB, syphilis (VDRL), and hepatitis B and C were negative. The cerebral spinal fluid (CSF) analysis was normal (**Table 2**). The chest computed tomography (CT) was normal, and the abdomen CT revealed hepatosplenomegaly.

**Table 1:** Laboratory exams.

Laboratory exams	Patient	Reference values
White-cell count (per mm³)	3600	3500 - 10500
Red blood cell count (per mm³)	3.1x10^6^	4.3x10^6^ - 5.70x10^6^
Hemoglobin (g/dl)	6.5	13.5 - 17.5
Hematocrit (%)	19.2%	39 - 50
V.C.M. (fL)	63	81 - 95
H.C.M. (pg)	21.3	26 - 34
C.H.C.M. (g/dl)	33.9	31 - 36
R.D.W. (%)	16.9	12- 15
Platelet count (per mm³)	262000	150000 - 450000
Band cells (per mm³)	3% (108 mm³)	0 - 840
Segmented (per mm³)	59% (2124 mm³)	1700 - 8000
Eosinophils (per mm³)	1% (36 mm³)	50-500
Lymphocytes (per mm³)	25% (900 mm³)	900 - 2900
Monocytes (per mm³)	12% (432 mm³)	300 - 900
Reticulocytes (%)	0.3	0.5-1.5
Serum iron (mcg/dL)	25.4	33-193
Total iron binding capacity (TIBC) (mcg/dL)	171.4	135-392
Transferrin saturation (%)	14.82	20-50
Ferritin (ng/mL)	732.50	30-400
Glucose (mg/dL)	76.8	70-99
Hemoglobin A1c (HbA1c) (%)	5.6	4-7
Creatinine (mg/dL)	2.22	0.70 - 1.20
Urea (mg/dL)	63.4	16.6-48.5
C-reactive protein (mg/dL)	5.28	<0.5
Gamma-glutamyl transferase (GGT) (U/L)	42	<60
AST (U/L)	45.8	10-50
ALT (U/L)	26	10-50
Alkaline phosphatase (U/L)	98	40-129 U/L
Albumin (g/dL)	2.75	3.97-4.95
TSH (mU/L)	1.44	0.270-4.20
T4 (ng/dL)	1.32	0.93-1.7
Folate (nmol/L)	4.69	4.6-34.8
Vitamin B12 (pg/mL)	534.4	211-946
25-hydroxyvitamin D (25(OH)D) (ng/mL)	3	30-60
Erythrocyte sedimentation rate (ESR) (mm/h)	69	<20
Serum potassium (mEq/L)	3.29	3.5-5.5
Serum calcium (mg/dL)	10.66	8.6-10
Serum magnesium (mg/dL)	1.83	1.6-2.6
Serum sodium (mEq/L)	137	134-145

**Table 2:** Cerebrospinal fluid analysis.

Cerebrospinal fluid		
	Patient	Reference values
Appearance	Clear	Clear
Glucose (mg/dL)	49.3	50 a 90
Protein (mg/dL)	24	15 - 45
White blood cells count (cel/mm³)	04	<5
Red blood cells count (cel/mm³)	01	-
Rapid molecular test for tuberculosis	Negative	Negative
Acid-fast Bacillus culture	Negative	Negative
Fungal culture	No growth	No growth
Direct microscopic examination of fungi	Negative	Negative
VDRL	Nonreactive	Nonreactive

Electroneuromyography showed an asymmetrical sensory and motor axonal neuropathy with focal slowing of conduction velocity and severe impairment of ulnar nerves in the elbow segment and tibial nerves in the ankle. Ultrasonography of peripheral nerves displayed echographic findings suggestive of hypertrophic neuropathy of the ulnar, median, left peroneal, and tibial nerves (more evident at pre-tunnel levels) in addition to signs of intraneural hyperemia in the left ulnar nerve at the pre-tunnel level and mild thickening of the right peroneal nerve.

**Figure 1:**
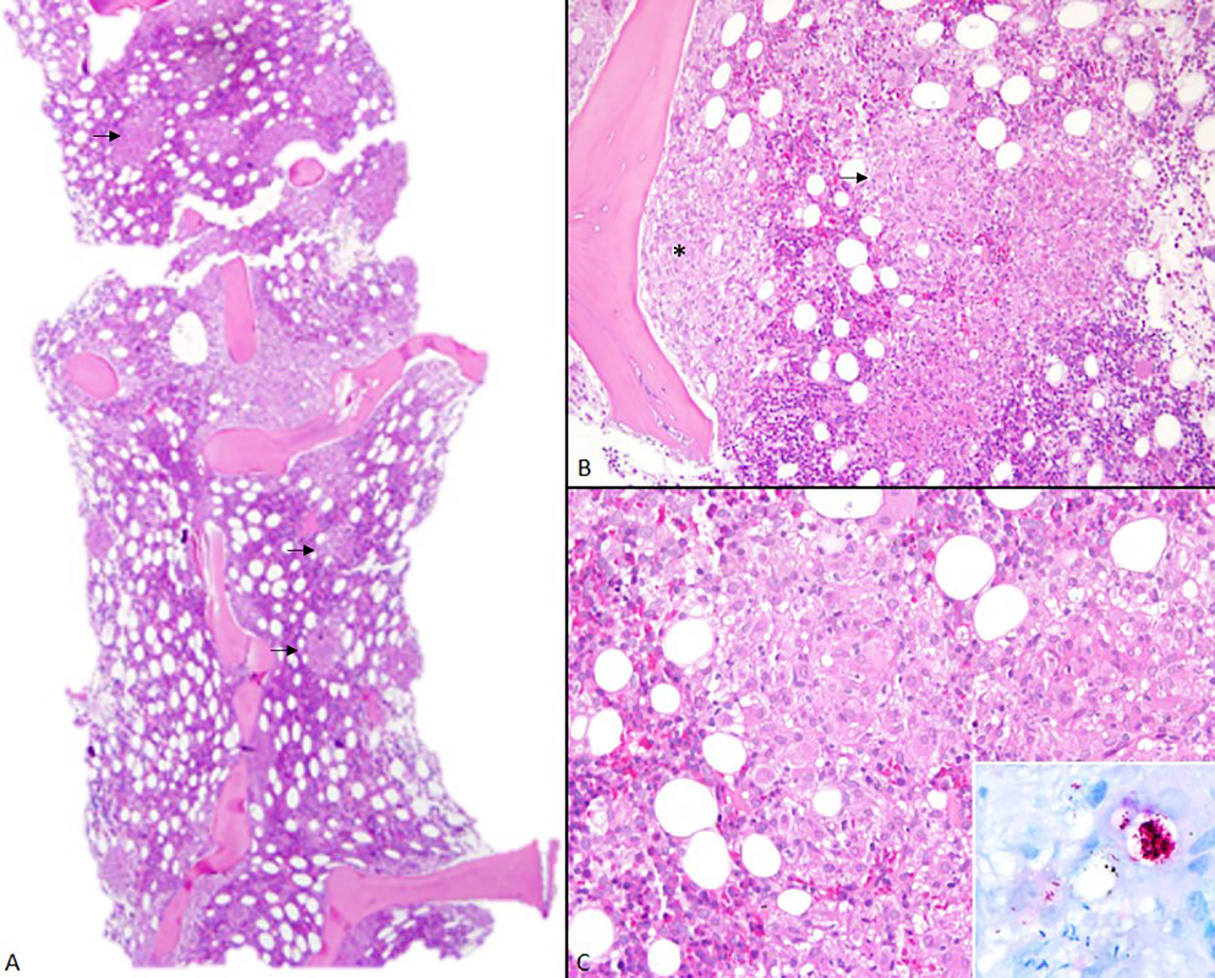
Bone marrow. **(A)** At low power, bone marrow microscopic examination shows hypercellularity in nodular pattern (arrows) (H&E, 2x). **(B)** Noncaseating epithelioid granulomas in interstitial (arrow) and paratrabecular loci (*) (H&E, 10x). **(C)** These granulomas are composed mostly of epithelioid macrophages and a few dispersed lymphocytes (20x, H&E). In detail, macrophages are enlarged by large collections of leprosy bacilli – globi (100x, Faraco-Fite).

**Figure 2:**
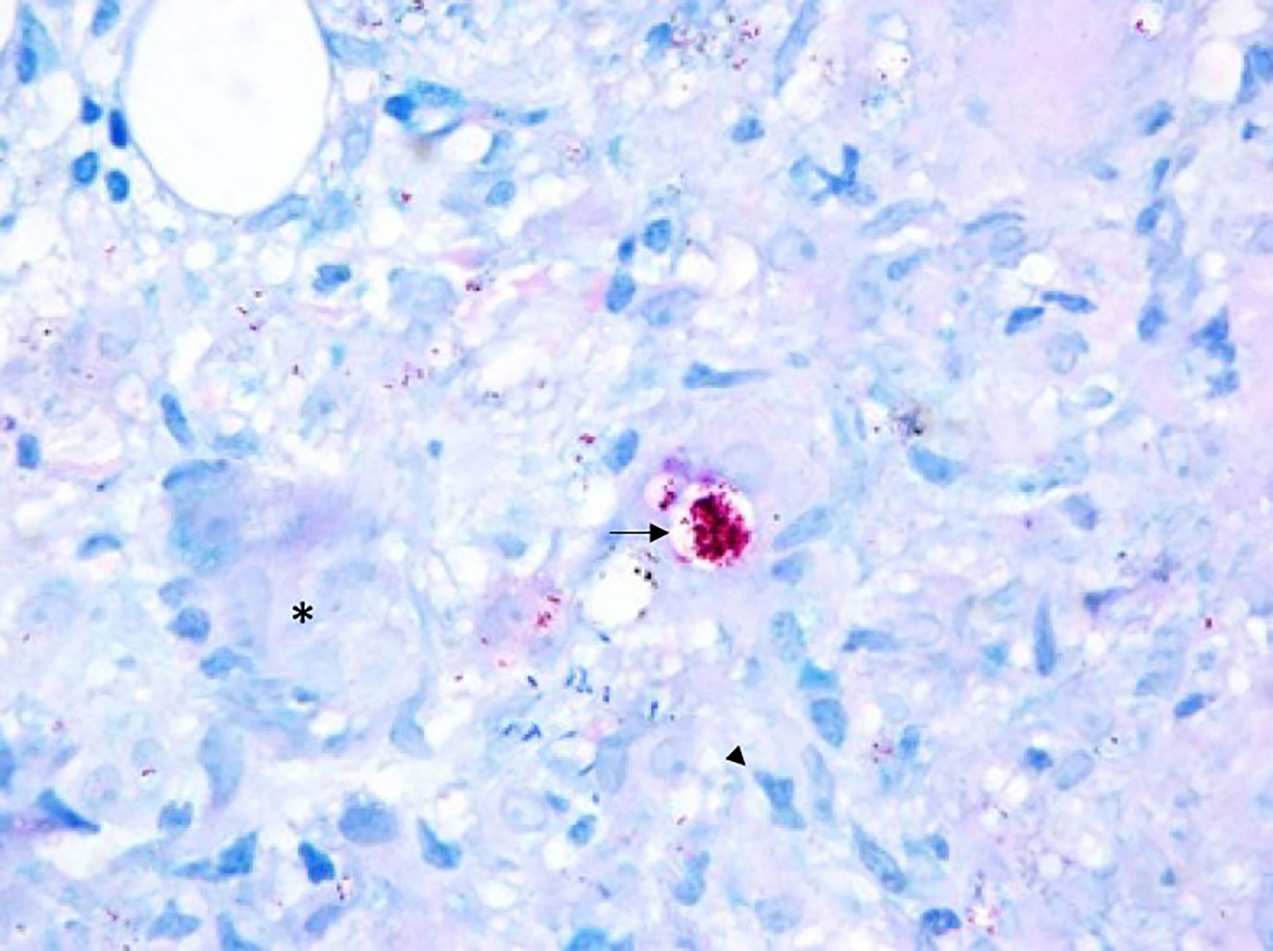
Bone Marrow - Epithelioid granuloma composed of macrophages (arrow head) and multinucleated giant cells (*) rich in bacilli, which may present in isolation or bound together forming globi (arrow), what distinguishes M. leprae from other mycobacteria (100x, Faraco-Fite).

A bone marrow biopsy (**Figures 1**, **2**) revealed the presence of *M. leprae* forming globi. The ELISA anti-phenolic glycolipid I (PGL-I) IgM antibodies and *M. leprae* DNA were positive, with index 2.38 (normal values: <1 - negative) (7). The slit skin smear Bacilloscopy Index (BI) mean was 4.16 (**Table 3**).

**Table 3:** Bacilloscopic index.

Location	Bacilloscopic index
Right ear lobe	4
Left ear lobe	5
Right elbow	4
Left elbow	4
Right knee	4
Left knee	4
**Bacilloscopy Index (BI) mean**	4.16

To detect *M. leprae* DNA, were used a real-time quantitative polymerase chain reaction (qPCR) primer/probe assay, described elsewhere, to target the *M. leprae* species-specific genomic element of dispersed repeats (RLEP) (ABI 7300 realtime PCR system; Applied Biosystems). The slit skin smear DNA *M. leprae* qPCR was positive Ct = 19 (normal values ≤ 35) with 3.8x10^8^ copies/mL. The skin biopsy showed whole bacilli with BI equal to 6.0 and Ct = 21 (normal values ≤ 35) with 2.1x10^9^ copies/gram. The diagnosis of lepromatous leprosy was made. The treatment was started with a supervised monthly dose of rifampin (600 mg), ofloxacin (400 mg), and minocycline (100 mg), and an unsupervised daily dose of ofloxacin (400 mg) and minocycline (100 mg). This treatment was chosen to avoid the use of dapsone, due to the possibility of drug-induced anemia worsening the patient’s condition.

During the first months of treatment, the patient gained weight, his nutritional status improved, and he was able to perform independent activities of daily living again. He has been followed up monthly by a multi-professional team at our outpatient clinic with good adherence to the proposed treatment.

## Discussion

The presence of *M. leprae* in bone marrow aspirates or biopsies was already described in previous case reports (5, 6). Even though major studies are necessary, some authors believe the presence of *M. leprae* in the bone marrow, especially in lepromatous leprosy, can act as a reservoir of the disease facilitating future relapses (8).

Although bone marrow involvement in leprosy is rare, it is important to investigate bone marrow involvement in leprosy, especially when the patient presents with cytopenias (9). The differential diagnosis can be broad and variable depending on the clinical presentation. The majority of the cases involve the investigation of FUO, wasting syndrome, and cytopenias. In those cases, the main differential diagnosis was lymphoproliferative diseases that were considered less probable after the laboratory results revealed a normal pattern for serum protein electrophoresis (SPEP) and the absence of concerning findings for neoplasia on the abdomen and chest computerized tomography (CT) (4–6).

Shepard and Karat (1972) used the inoculation of mice to show bacilli in the bone marrow were viable and able to multiply. They hypothesized that the presence of *M. leprae* in the bone marrow could lead to inhibition of lymphocytes’ sensitivity for the mycobacteria, which diminishes the immune response in peripheral tissues for those with lepromatous leprosy (10).

Karat (1975) used footpads of mice to show that *M. leprae* remained viable in the bone marrow long after it ceased to be viable in the skin following treatment with oral dapsone or clofazimine (11). Since that time, leprosy treatment has evolved despite a lack of studies to clarify the relationship of the bacilli with the immune system and its presence in the bone marrow. The high number of leprosy relapses raises concern for a possible mechanism involving the persistence of bacilli multiplying in organs, such as bone marrow, after the standard treatment, especially in lepromatous leprosy (8, 12, 13).

The patient also developed cachexia, another contributor to the severe weakness presented by the patient. The weight loss in leprosy can be associated with the inflammatory state, especially during reactional episodes. High levels of tumor necrosis factor-alpha (TNF-α), interleukin-1 (IL-1), interferon-gamma (IFN-γ), and other cytokines are found in leprosy patients during reactional episodes (14, 15). Those same cytokines are related to the mechanisms of muscle wasting related to cachexia and weight loss (16). Besides that, TNF-α and IFN-γ may be involved in the pathogenesis of peripheral neuropathy (17), as seen in the severe neuropathy presented by our patient.

The laboratory exam revealed a remarkably low vitamin D level. There are studies associating vitamin D receptor (VDR) polymorphism with leprosy (18–20), but robust studies correlating serum vitamin D level, receptor polymorphism, and response to *M. leprae* are still needed. However, in this case, the low level of vitamin D can also be a consequence explained by the patient being bedridden without sun exposure, which is an important factor in the conversion of this vitamin. Furthermore, the cachexia, confirmed by observing the serum albumin and folate, may contribute to the low vitamin D.

Although a liver biopsy was not performed, we believe that hepatosplenomegaly was caused by lepromatous leprosy. It was either caused by the direct presence of the *M. leprae* or as part of an immune reaction to the pathogen. Previous studies have already shown leprosy as a cause of granulomatous hepatitis (21–25). Hepatosplenomegaly can also be present during a reaction episode showing a different histopathological pattern, similar to the one present on the reactionary erythema nodosum on the skin (25). The hepatic involvement in leprosy is usually with absent or mild increase on liver function tests, especially aminotransferases, but a previous case report shows the possibility of high serum aminotransferase levels from leprosy-induced liver disease (24, 25).

Another important aspect of this case is the delay in the diagnosis. The patient was evaluated at different points of the healthcare network without a previous diagnosis. The delay in diagnosis can be partly explained by the lack of appreciation of the family history of leprosy (mother and brother) as a risk factor for the patient’s illness. Evidence shows increased risk for developing leprosy among household contacts, especially for exposure to contacts diagnosed with multibacillary disease and older patients (26). Ideally, this patient should have been monitored since the first diagnosis of leprosy in his household. Brazilian guidelines emphasize the importance of epidemiological vigilance and surveillance of household and social contacts. It is suggested that the contacts should be evaluated every year for at least 5 years (27).

Unfortunately, leprosy has not been listed often among the differential diagnoses of FUO in the literature (28), which likely contributed to the delay in the diagnosis and clinical management of our patient. Therefore, leprosy should be considered in the differential diagnosis of FUO associated with neuropathies and wasting syndrome. The case shows the importance of the follow-up and active search for cases in household contacts. In this case, an early diagnosis could have prevented major sequelae and severe disease.

## Data Availability Statement

The raw data supporting the conclusions of this article will be made available by the authors, without undue reservation.

## Ethics Statement

The studies involving human participants were reviewed and approved by the local research ethics committee CAAE: 57585422200005152. The patients/participants provided their written informed consent to participate in this study. Written informed consent was obtained from the individual(s) for the publication of any potentially identifiable images or data included in this article.

## Author Contributions

MS, WC, BD, and IG conceived of the presented idea. MS, IG, WC, and IC contributed to the data collection. MS and IG took the lead in writing the manuscript with support from all the other authors. All authors contributed to manuscript revisions. All authors contributed to the final approval of the version to be published.

## Funding

This study received funding from: FAPEMIG (Research Support Foundation of the State of Minas Gerais, Grant number: APQ - 02810-21) and the National Health Fund - Ministry of Health of Brazil. Grant number: TED 123/DIPOC 003/2020.

## Conflict of Interest

The authors declare that the research was conducted in the absence of any commercial or financial relationships that could be construed as a potential conflict of interest.

## Publisher’s Note

All claims expressed in this article are solely those of the authors and do not necessarily represent those of their affiliated organizations, or those of the publisher, the editors and the reviewers. Any product that may be evaluated in this article, or claim that may be made by its manufacturer, is not guaranteed or endorsed by the publisher.
